# New genera of Australian stiletto flies (Diptera, Therevidae)

**DOI:** 10.3897/zookeys.618.8059

**Published:** 2016-09-19

**Authors:** Michael E. Irwin, Shaun L. Winterton

**Affiliations:** 1 Illinois Natural History Survey, Champaign, Illinois USA; 2 California State Collection of Arthropods, California Department of Food & Agriculture, Sacramento, California, USA

**Keywords:** Stiletto fly, Therevidae, Therevoid clade, Asiloidea, Australia

## Abstract

Two new stiletto fly genera of Agapophytinae (Diptera: Therevidae) are described from Australia. *Sidarena* **gen. n.** comprises six new species (*Sidarena aurantia* **sp. n.**, *Sidarena flavipalpa* **sp. n.**, *Sidarena geraldton* **sp. n.**, *Sidarena hortorum* **sp. n.**, *Sidarena macfarlandi* **sp. n.**, and *Sidarena yallingup* **sp. n.**) and is largely endemic to Western Australia. *Zelothrix* gen. n. is described based on two species; *Zelothrix warrumbungles* **sp. n.** is a locally abundant species in Eastern Australia, while *Zelothrix yeatesi* **sp. n.** is restricted to southwestern Western Australia. These sister genera are likely closely related to *Taenogerella* Winterton & Irwin and *Actenomeros* Winterton & Irwin.

## Introduction

The stiletto fly (Diptera: Therevidae) fauna of Australasia is the most species-rich biogeographical region, comprising over 400 described species in 26 genera. Two of the four subfamilies of therevidae are present in Australasia, Agapophytinae (209 species in 23 genera) and Therevinae (166 spp. in 3 gen.) (Winterton 2009, 2011; Winterton et al. 2016); Xestomyzinae and Phycusinae (previously Phycinae) (Gaimari et al. 2013; ICZN 2015) are entirely absent from the region. All agapophytine genera and all but one therevine genus (i.e., *Irwiniella* Lyneborg, 1976) are endemic to Australasia.

**Figure 1.**
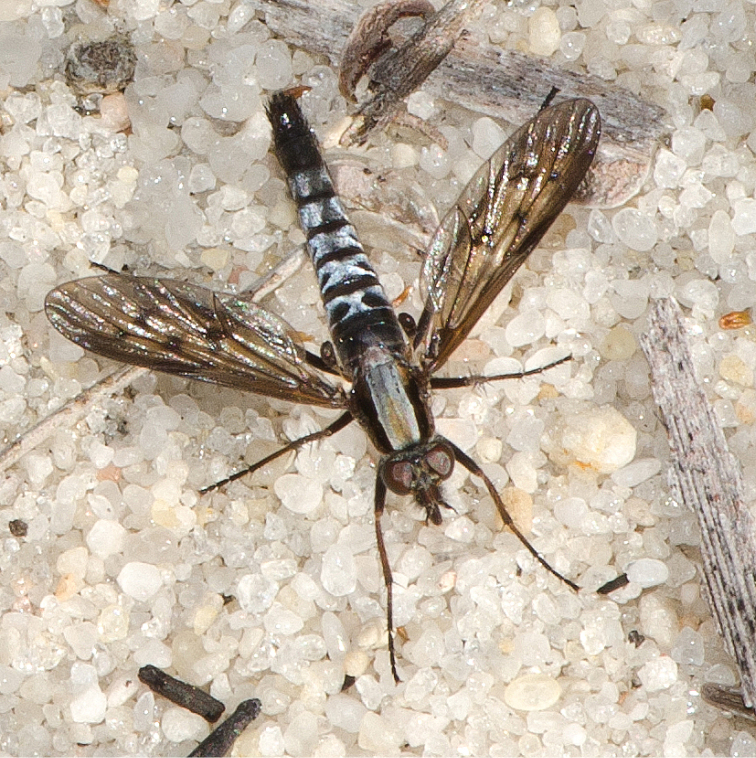
Adult male *Sidarena hortorum* sp. n.; Talbot Road Nature Reserve, Stratton, Perth, Western Australia. (Photo credit: Fred and Jean Hort).

**Figure 2.**
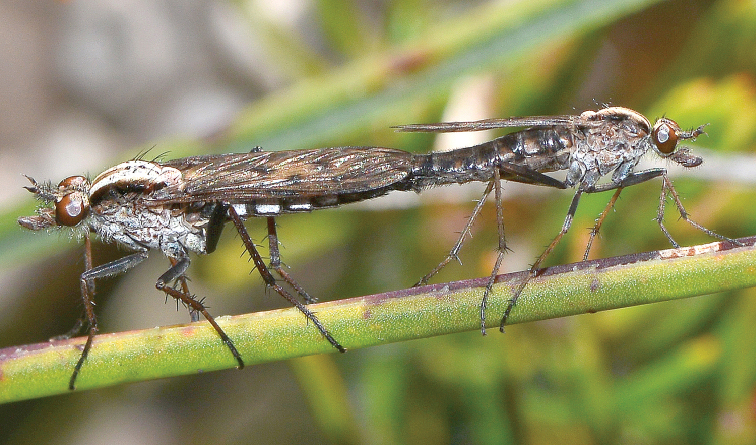
*Sidarena hortorum* sp. n. mating pair; Bullsbrook Nature Reserve, Bullsbrook, Western Australia. (Photo credit: Fred and Jean Hort).

**Figure 3.**
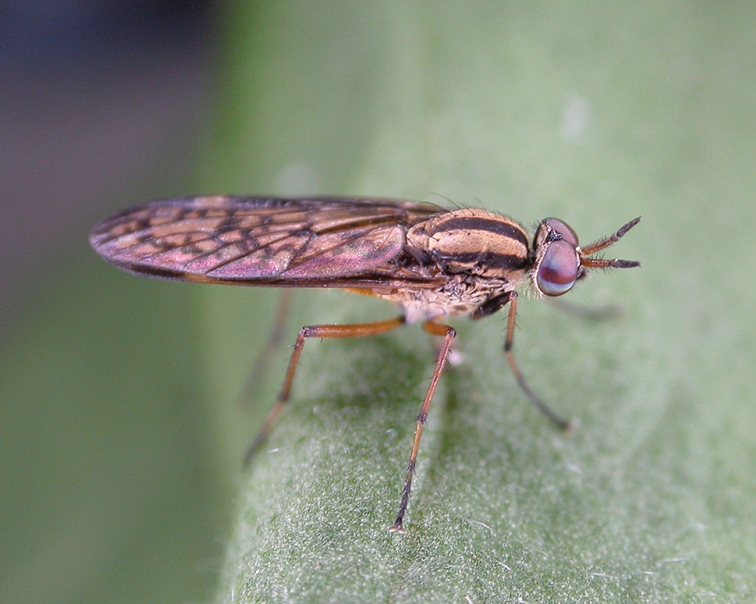
Adult male *Zelothrix yeatesi* sp. n.; Warrumbungle National Park, New South Wales. (Photo credit: Shaun L. Winterton).

Numerous publications describing new subfamilies, genera and species of Australian therevids have been published over the last 10 years (e.g., Ferguson et al. 2013, 2014; Winterton 2007a-d, 2009, 2011a, b; Winterton and Ferguson 2012; Winterton and Lambkin 2012; Lambkin and Turco 2013) including overarching phylogenies of the family by Lambkin et al. (2009) and Winterton et al. (2016), yet new genera and species continue to be discovered and described. Herein we describe two new genera of agapophytine therevids from Australia, *Sidarena* gen. n. (Figs 1–2) and *Zelothrix* gen. n. (Fig. 3). Both genera were recovered as sister groups (both identified as ‘undescribed genus S’) in the recent paper on therevid phylogeny by Winterton et al. (2016) and appear closely related to genera such as *Taenogerella* Winterton & Irwin and *Actenomeros* Winterton & Irwin. The new genera are diagnosed and keys to species are presented for each.

## Materials and methods

Adult morphological terminology follows Cumming and Wood (2009) with genitalic morphology as modified by Winterton et al. (1999a,b) and Winterton (2006). Genitalia were macerated in 10% KOH to remove soft tissue, then rinsed in distilled water and dilute glacial acetic acid, and dissected in 80% ethanol. Genitalia preparations were placed in glycerine in a genitalia vial mounted on the pin beneath the specimen.

Specimen images were taken at different focal points using a digital camera and subsequently combined into a serial montage image using Helicon Focus software. All new nomenclatural acts are to be registered in ZooBank (Pyle and Michel 2008). Types are deposited in the following institutions and collections: Australian National Insect Collection (Canberra) (ANIC), Western Australian Museum (Perth) (WAM), California Academy of Sciences (San Francisco) (CAS), California State Collection of Arthropods (Sacramento) (CSCA), Canadian National Insect Collection (Ottawa) (CNC), Queensland Museum (Brisbane) (QM). Numbers quoted with individual specimens as MEI000000 are unique identifiers in the therevid database MANDALA and are attached to each specimen as a yellow or white label (Kampmeier and Irwin 2009). Material examined lists were exported from MANDALA. Abbreviations in text: notopleural setae (np); supra alar setae (sa); postalar setae (pa); dorsocentral setae (dc); scutellar setae (sc).

## Taxonomy

### Sidarena gen. n.

Taxon classificationAnimaliaDipteraTherevidae

http://zoobank.org/CCC28E9C-EB5D-4776-A269-770CFDE5FBF0

Figs 1 , 2 , 5 , 6 , 7 , 8 , 9 , 10 , 11 , 12 , 13 , 14 , 15 , 16 , 17 , 18 , 19 , 20 , 28B

#### Type species.

*Sidarena macfarlandi* sp. n., designated here.

#### Diagnosis.

Both sexes with eyes widely dichoptic; multiple poorly defined rows of postocular macrosetae present dorsally in both sexes; antennal scape lacking macrosetae along medial surface, scape shorter then head length; flagellum conical, tapering to a terminal arista; parafacial setae absent; one pair of scutellar macrosetae, most other scutal macrosetae variable in number; velutum patches absent on femora and sparsely present ventrally on gonocoxites; single anteroventral seta present apically on hind femur; wing slightly to dark infuscate with maculae, cell m_3_ open to wing margin; abdomen narrow elongate; male abdomen typically with silver velutum; male genitalia with articulating inner gonocoxal process well developed; outer gonocoxal process well developed and rounded; gonocoxal apodeme short and rounded; gonostylus narrow apically; gonocoxites lacking medial atrium; aedeagus with distiphallus broad apically, not directed ventrally at apex; epandrium shape quadrangular; ventral apodeme of parameral sheath forked. Female tergite 8 with broad anteromedial process; three spermathecae, ducts joining to spermathecal sac duct; spermathecal sac present (Fig. 28B).

#### Included species.

*Sidarena aurantia* sp. n., *Sidarena flavipalpa* sp. n., *Sidarena geraldton* sp. n., *Sidarena hortorum* sp. n., *Sidarena macfarlandi* sp. n., and *Sidarena yallingup* sp. n.

#### Comments.

*Sidarena* gen. n. is mostly endemic to Western Australia and is distinctive in general appearance, with male eyes widely dichoptic, numerous bristles on the occiput and often with grey metallic pubescent stripe medially on the scutum (often adjoining broad matte black-brown pubescent stripes laterally). The presence of these characters alone differentiates this genus from all other genera in the subfamily. Similar genera to *Sidarena* gen. n. include *Squamopygia* Kröber, *Ectinorhynchus* Macquart and *Zelothrix* gen. n. The new genus can be quickly differentiated from *Squamopygia* by the much shorter scape (narrowly elongate cylindrical in *Squamopygia*), and the wing not distinctly banded (two black bands in *Squamopygia*). *Sidarena* gen. n. is separable from *Ectinorhynchus* by the absence of a medial atrium in the male gonocoxites (present in *Ectinorhynchus*) and separable from *Zelothrix* gen. n. by a single scutellar macroseta (two macrosetae in *Zelothrix* gen. n.) and three spermathecae (two in *Zelothrix* gen. n.). The male genitalia are remarkably uniform throughout the genus. A single species is herein described from Queensland while the remaining species are from Western Australia.

#### Etymology.

The genus name is derived from the Greek *Sideros*, meaning iron, referring to the broad metallic-grey stripe typically present on the thorax; and *arena*, referring to its habit of landing in sandy patches. Gender is feminine.

#### Key to species of *Sidarena* gen. n.

**Table:** 

1	Abdomen predominantly bright orange to dark yellow (Figs 5, 14)	**2**
–	Abdomen predominantly dark brown, often with yellow laterally (e.g., Figs 10, 12)	**3**
2	Abdominal tergite 1 uniformly orange; femora with extensive black suffusion, hind femur mostly brown to black; pubescence on lower half of pleuron and coxae sparse (Fig. 5)	***Sidarena aurantia* sp. n.**
–	Abdominal tergite 1 dark anteriorly; femora mostly orange with brown black suffusion evident only on hind femur; pubescence on lower half of pleuron and coxae relatively dense (Figs 14–15)	***Sidarena macfarlandi* sp. n.**
3	Femora dark yellow (Eastern Australia) (Figs 7–8)	***Sidarena flavipalpa* sp. n.**
–	Femora brown to dark brown (Figs 10, 12, 17) (Western Australia)	**4**
4	Wing distinctly mottled; six to eight dorsocentral macrosetae (Fig. 17)	***Sidarena yallingup* sp. n.**
–	Wing with relatively few faint markings; fewer than six large dorsocentral macrosetae (Fig. 10)	**5**
5	Scape largely greyish pubescent; abdomen base colour mostly blackish-brown; smaller species with wing relatively narrow (body length = 5.5 mm) (Fig. 10)	***Sidarena geraldton* sp. n.**
–	Scape largely cream-yellow pubescent (sometimes brown on lateral surface); abdomen base colour only brown dorsally, yellow laterally; larger species; wing relatively broad (body length = 8–9 mm) (Figs 12–13)	***Sidarena hortorum* sp. n.**

**Figure 4.**
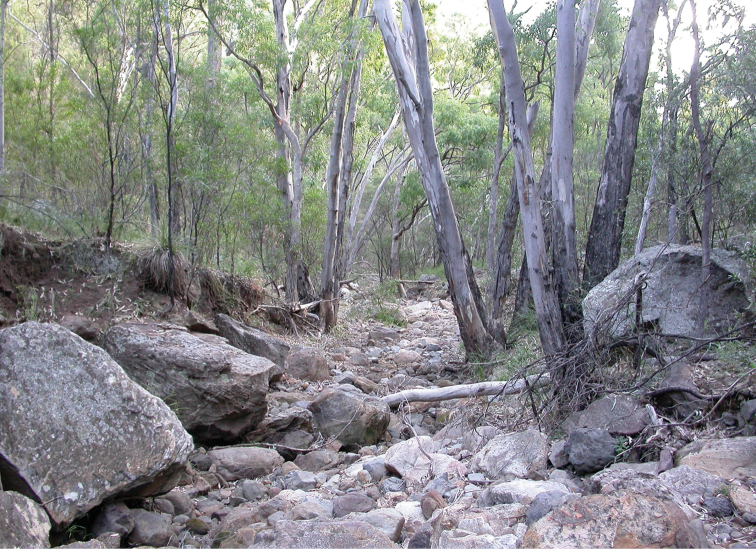
Warrumbungle National Park (New South Wales, Australia), creek bed in dry sclerophyll forest. Habitat of *Zelothrix warrumbungles* sp. n. where large numbers of individuals may be present during the summer months (Photo credit: Shaun L. Winterton).

**Figure 5.**
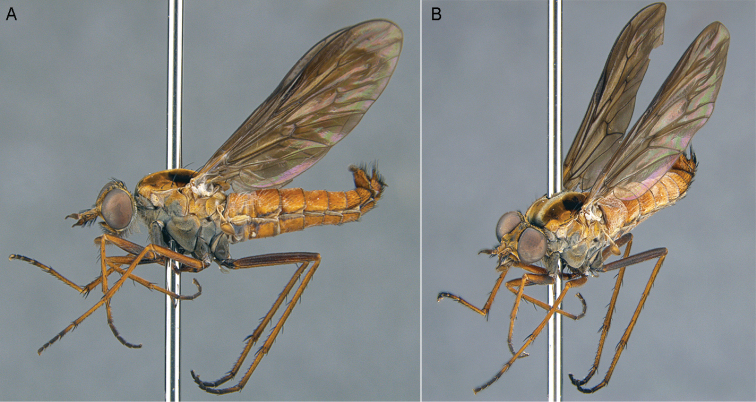
*Sidarena aurantia* sp. n.: **A** Male lateral view **B** same, oblique view.

**Figure 6.**
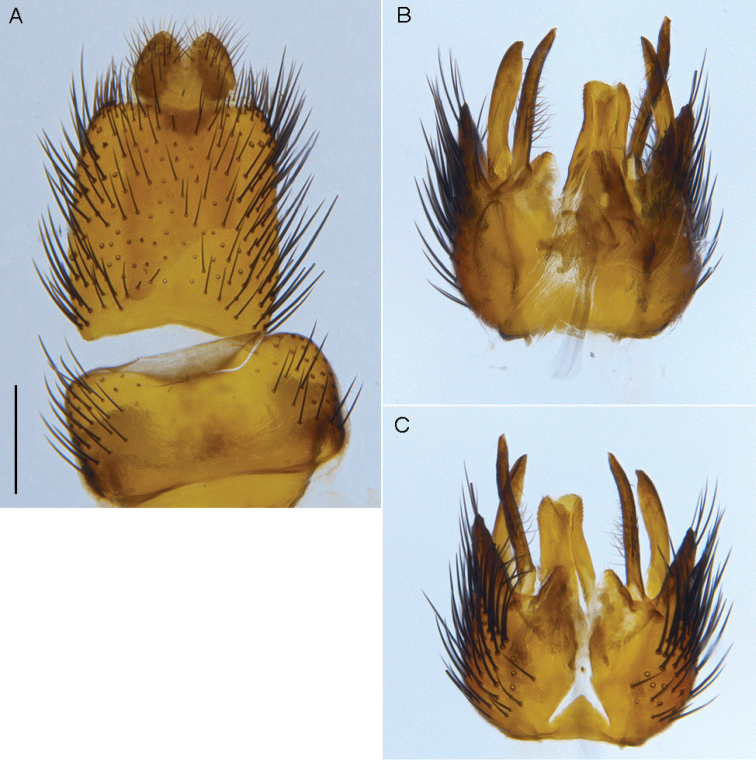
*Sidarena aurantia* sp. n., male genitalia: **A** Epandrium and tergite 8, dorsal view **B** Gonocoxites and aedeagus, dorsal view (epandrium removed) **C** same, ventral view. Scale line: 0.2 mm.

**Figure 7.**
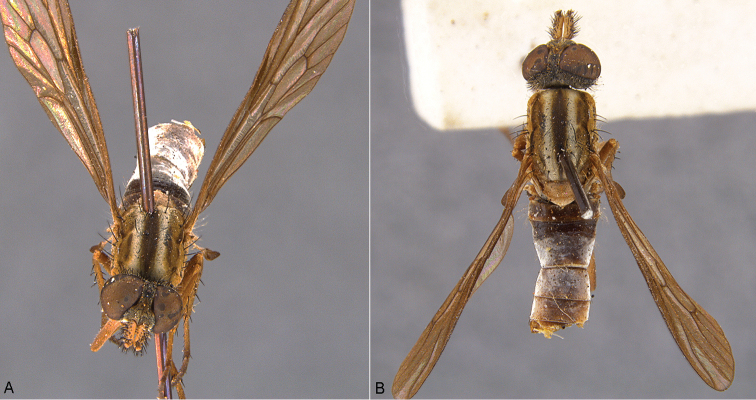
*Sidarena flavipalpa* sp. n.: **A** Male, anterior view **B** same, dorsal view.

**Figure 8.**
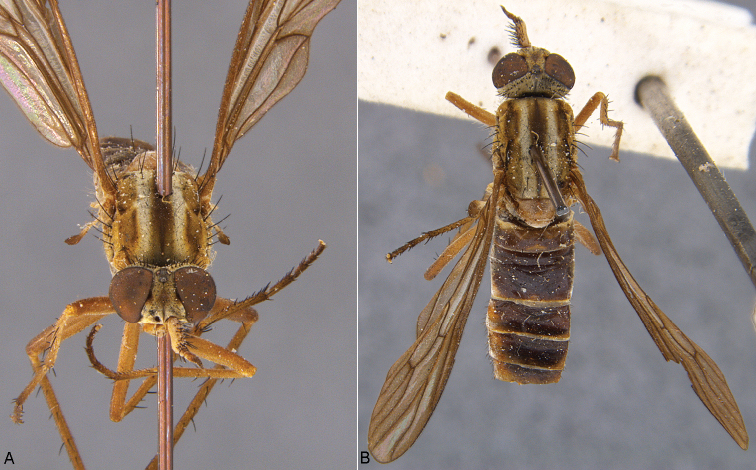
*Sidarena flavipalpa* sp. n.: **A** Female, anterior view **B** same, dorsal view.

**Figure 9.**
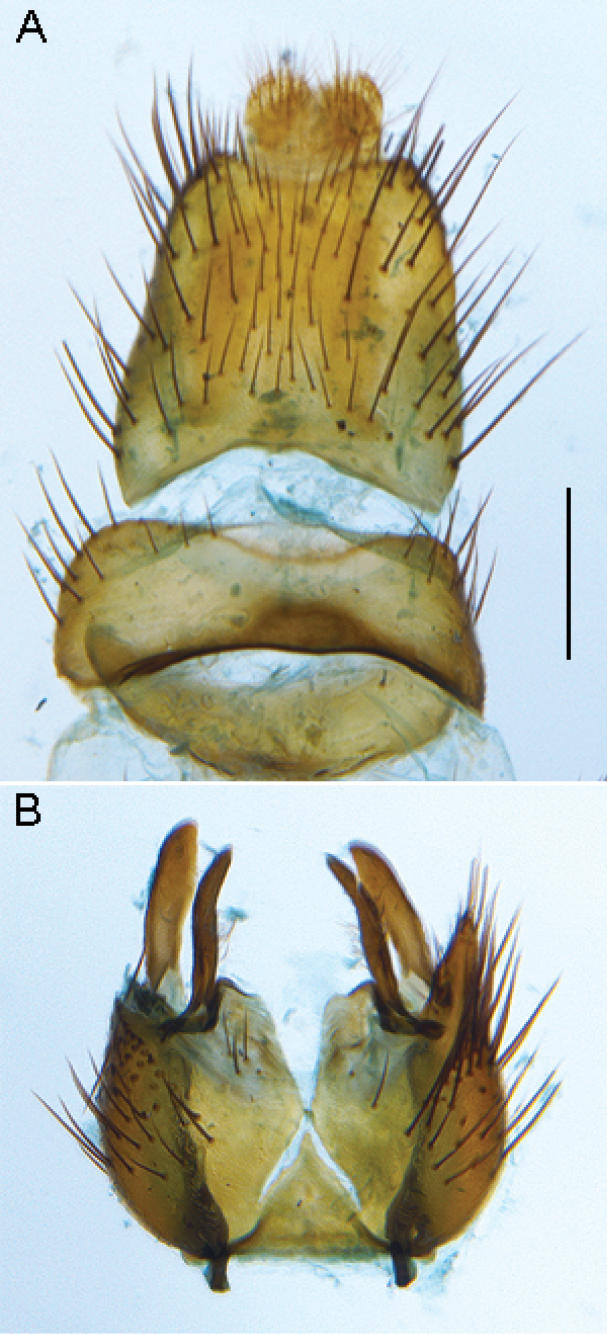
*Sidarena flavipalpa* sp. n.: **A** Epandrium and tergite 8, dorsal view **B** Gonocoxites, ventral view (epandrium and aedeagus removed). Scale line: 0.2 mm.

**Figure 10.**
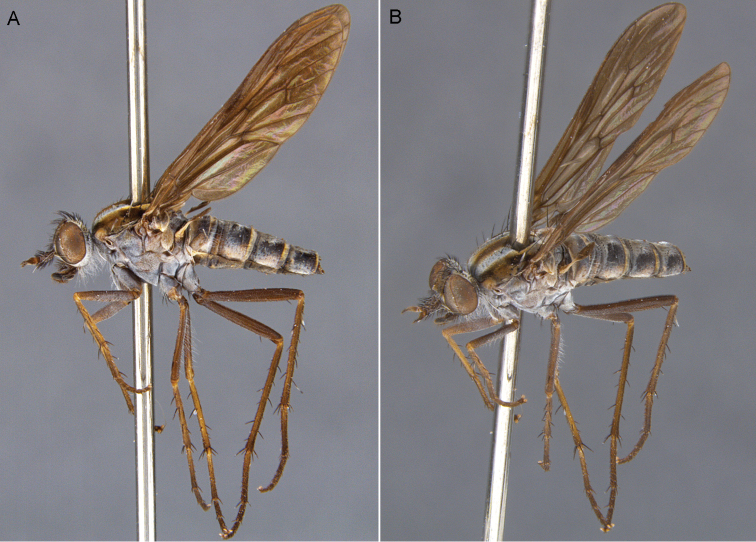
*Sidarena geraldton* sp. n.: **A** Male lateral view **B** same, oblique view.

**Figure 11.**
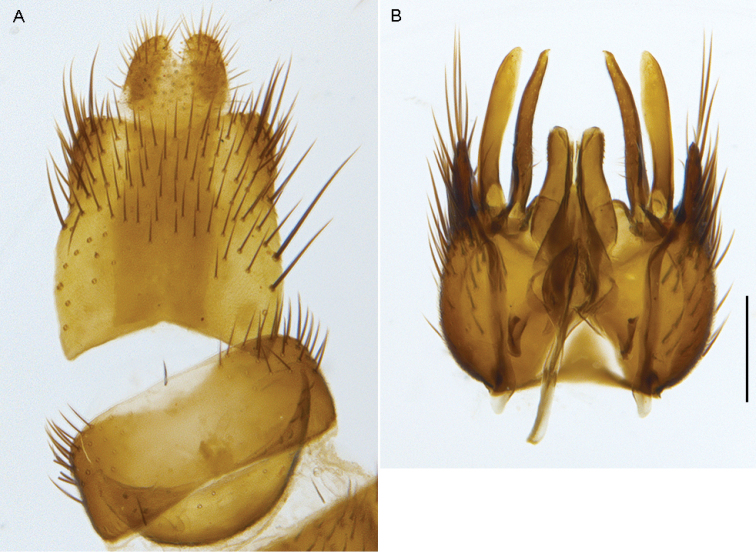
*Sidarena geraldton* sp. n., male genitalia: **A** Epandrium and tergite 8, dorsal view **B** Gonocoxites and aedeagus, dorsal view (epandrium removed). Scale line: 0.2 mm.

**Figure 12.**
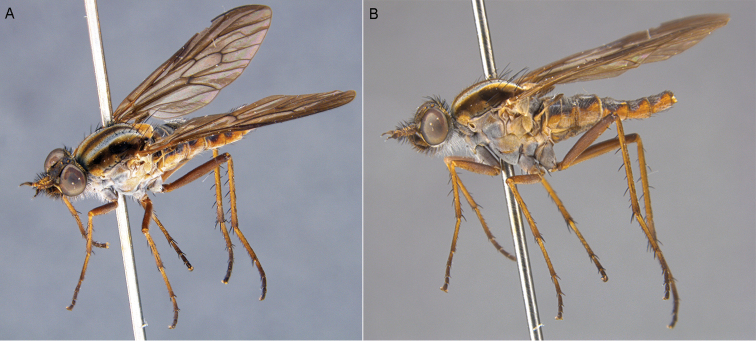
*Sidarena hortorum* sp. n.: **A** Male oblique view **B** same, lateral view (terminalia removed).

**Figure 13.**
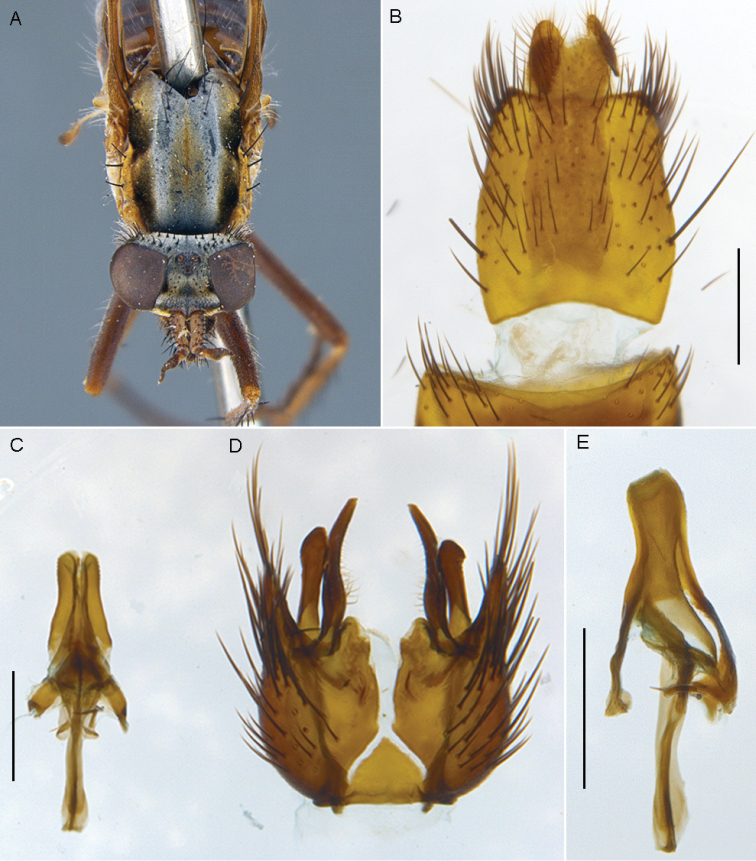
*Sidarena hortorum* sp. n.: **A** Female, head and thorax, dorsal view; male genitalia **B** Epandrium and tergite 8, dorsal view **C** Aedeagus, dorsal view **D** Gonocoxites, ventral view (epandrium and aedeagus removed) **E** Aedeagus, lateral view. Scale line: 0.2 mm.

**Figure 14.**
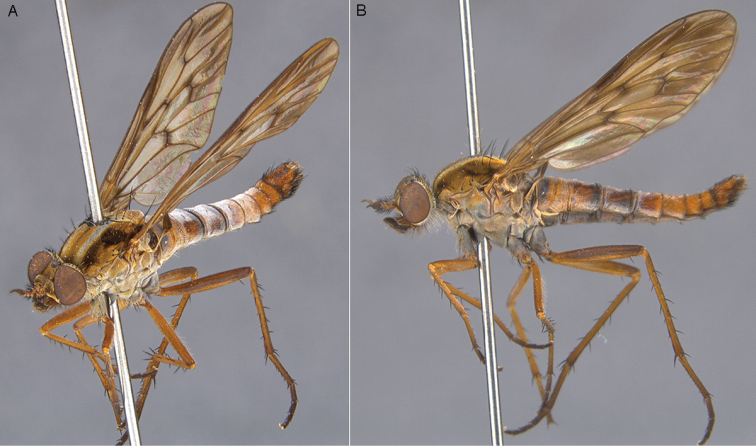
*Sidarena macfarlandi* sp. n.: **A** Male oblique view **B** same, lateral view.

**Figure 15.**
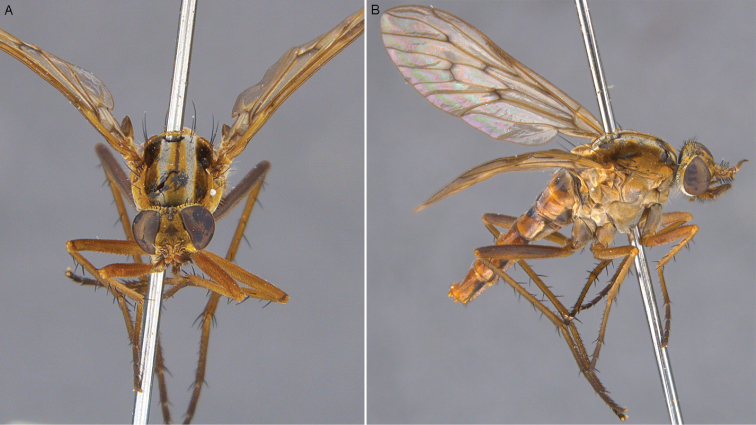
*Sidarena macfarlandi* sp. n.: **A** Female anterior view **B** same, lateral view.

**Figure 16.**
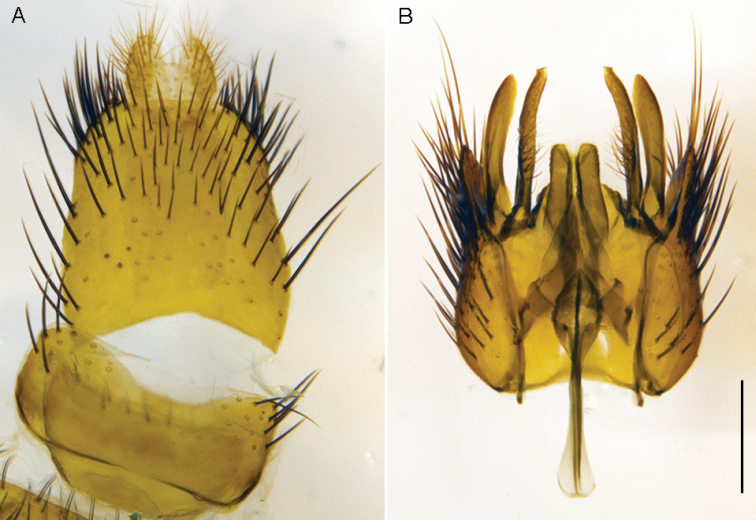
*Sidarena macfarlandi* sp. n., male genitalia: **A** Epandrium and tergite 8, dorsal view **B** Gonocoxites and aedeagus, dorsal view (epandrium removed). Scale line: 0.2 mm.

**Figure 17.**
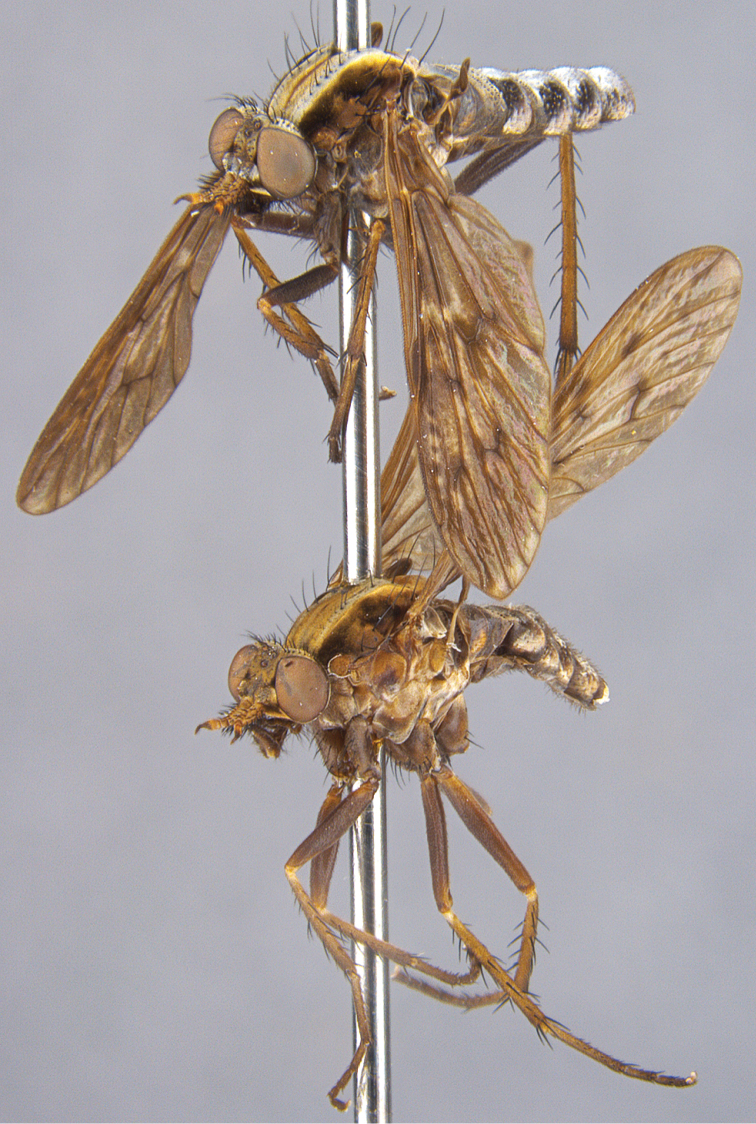
*Sidarena yallingup* sp. n.: Male (upper) and female (lower), oblique view (terminalia removed).

**Figure 18.**
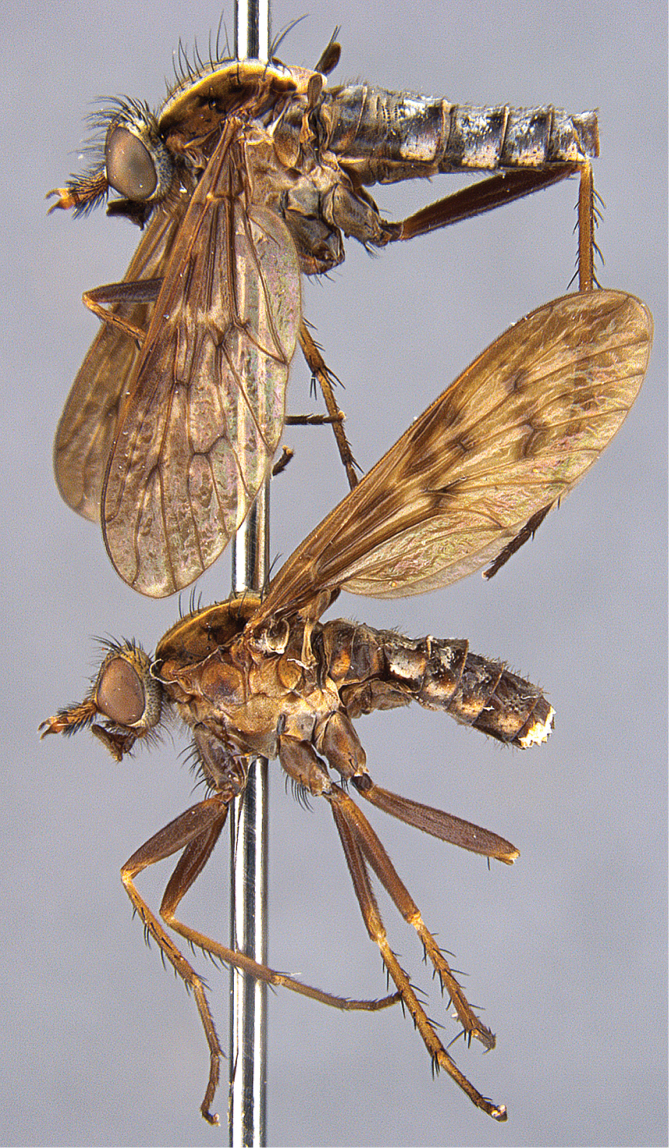
*Sidarena yallingup* sp. n.: Male (upper) and female (lower), lateral view (terminalia removed).

**Figure 19.**
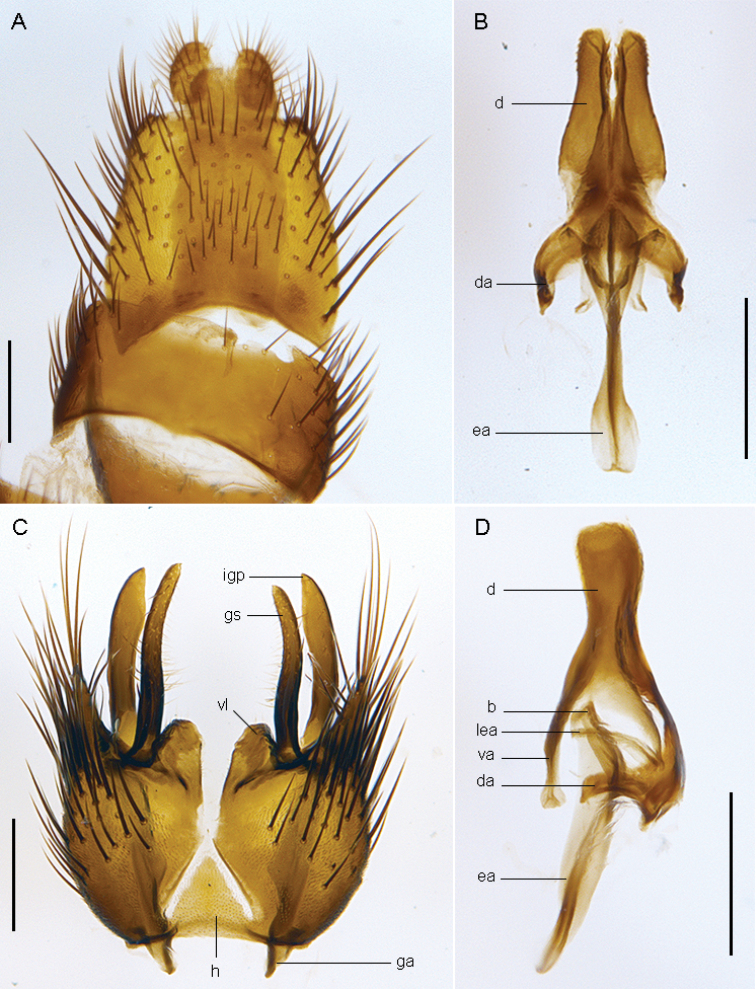
*Sidarena yallingup* sp. n.: Male genitalia: **A** Epandrium and tergite 8, dorsal view **B** Aedeagus, dorsal view **C** Gonocoxites, ventral view (epandrium and aedeagus removed) **D** Aedeagus, lateral view. Scale line: 0.2 mm. Abbreviations: *b*, basiphallus; *d*, distiphallus; *da*, dorsal apodeme of parameral sheath; *ea*, ejaculatory apodeme; *ga*, gonocoxal apodeme; *gs*, gonostylus; *h*, hypandrium; *igp*, inner gonocoxal process; *lea*, lateral ejaculatory apodeme; *va*, ventral apodeme of parameral sheath; *vl*, ventral lobe. Scale line = 0.2 mm.

**Figure 20.**
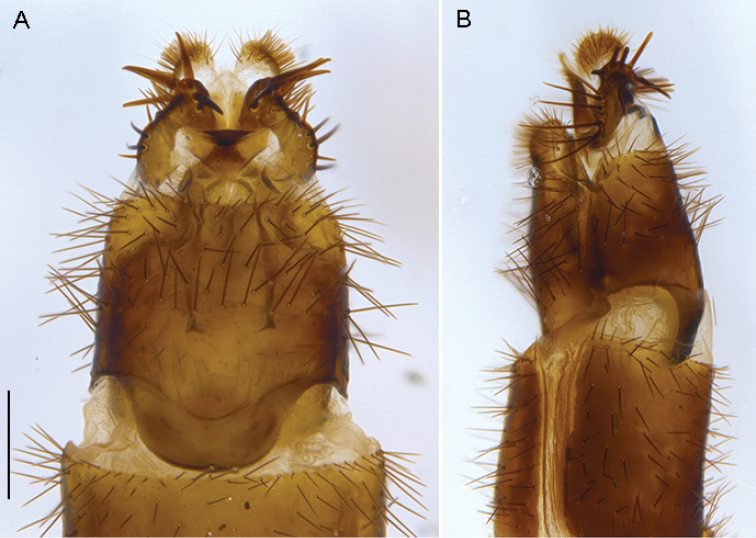
*Sidarena yallingup* sp. n.: Female genitalia: **A** dorsal view **B** lateral view. Scale line: 0.2 mm.

**Figure 21.**
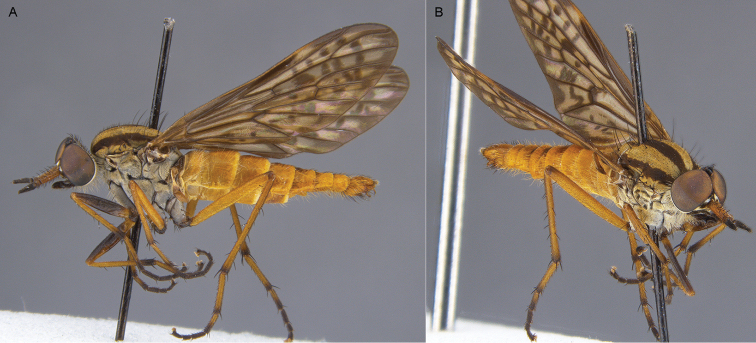
*Zelothrix warrumbungles* sp. n.: **A** Male lateral view **B** same, oblique view.

**Figure 22.**
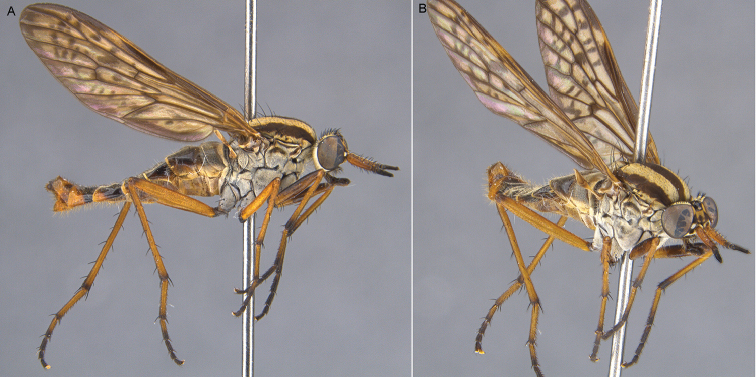
*Zelothrix warrumbungles* sp. n.: **A** Female lateral view **B** same, oblique view.

**Figure 23.**
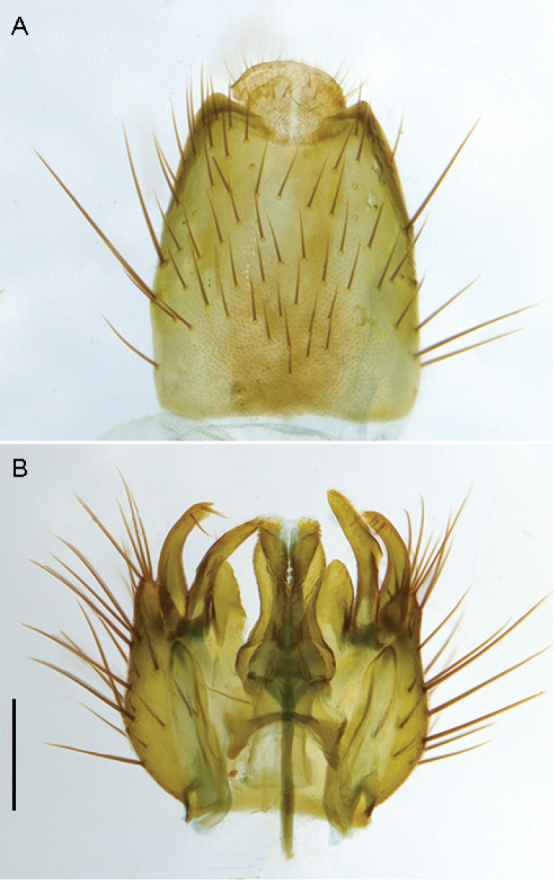
*Zelothrix warrumbungles* sp. n.: Male genitalia: **A** Epandrium, dorsal view **B** Gonocoxites and aedeagus, dorsal view (epandrium removed). Scale line: 0.2 mm.

**Figure 24.**
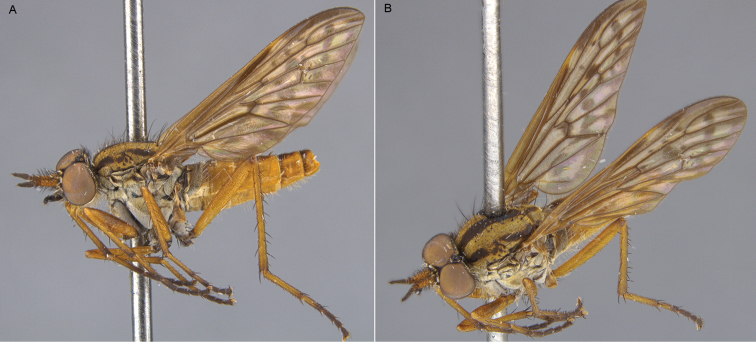
*Zelothrix yeatesi* sp. n.: **A** Male lateral view **B** same, oblique view (terminalia removed).

**Figure 25.**
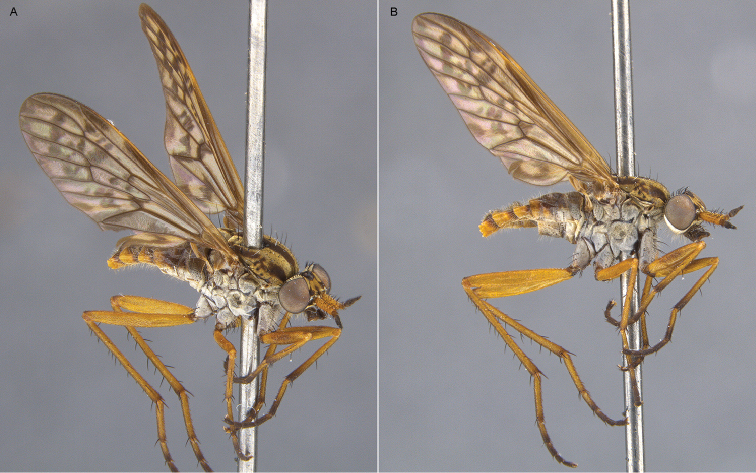
*Zelothrix yeatesi* sp. n.: **A** Female oblique view **B** same, lateral view (terminalia removed).

**Figure 26.**
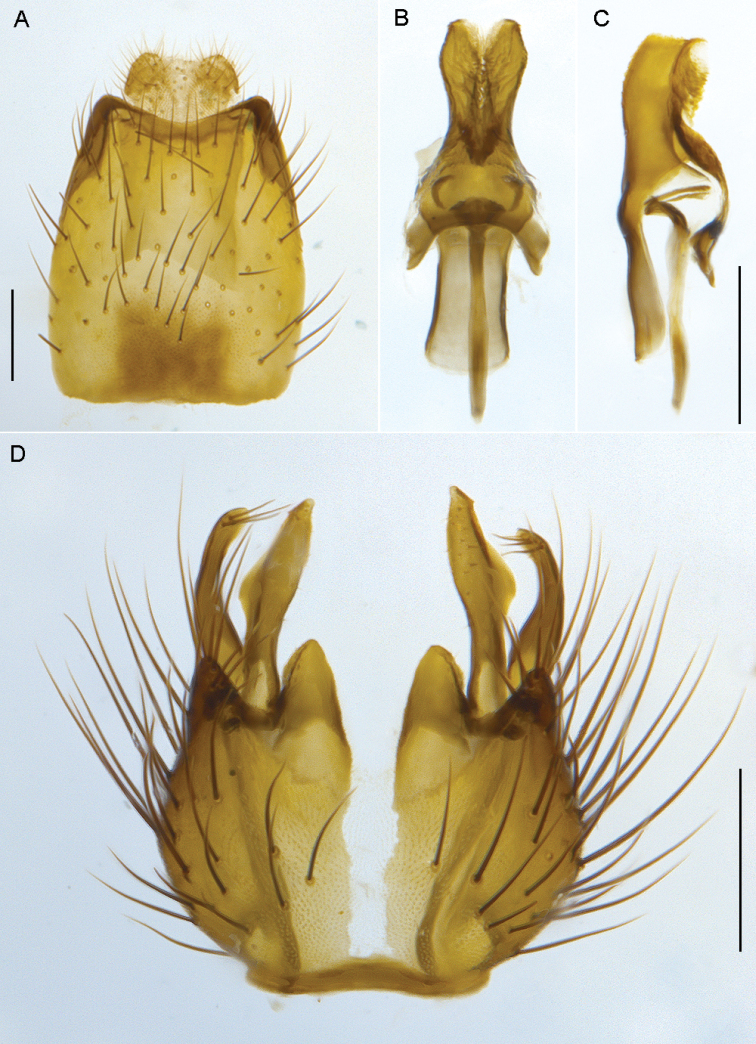
*Zelothrix yeatesi* sp. n.: Male genitalia: **A** Epandrium, dorsal view **B** Aedeagus, dorsal view **C** Same, lateral view **D** Gonocoxites, ventral view (epandrium and aedeagus removed). Scale line: 0.2 mm.

**Figure 27.**
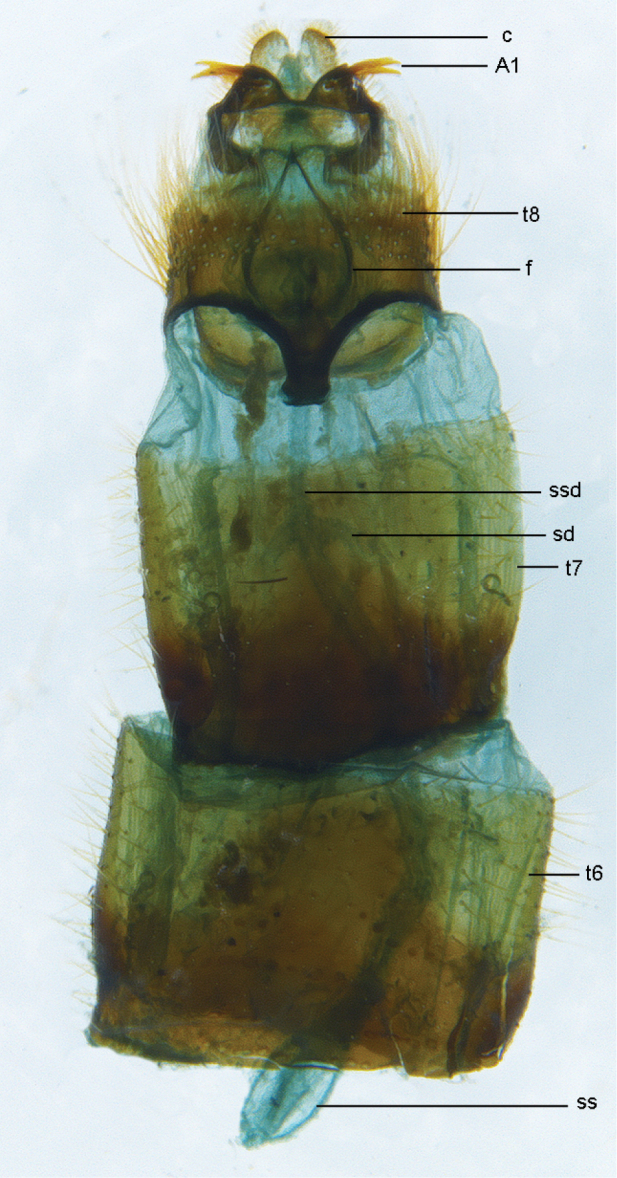
*Zelothrix yeatesi* sp. n.: Female genitalia, dorsal view. Abbreviations: c, cercus; A1 acanthophorite spines A1; t6–t8; tergites 6–8; *sd*, spermathecal duct; *ss*, spermathecal sac; *ssd*, spermathecal sac duct.

**Figure 28.**
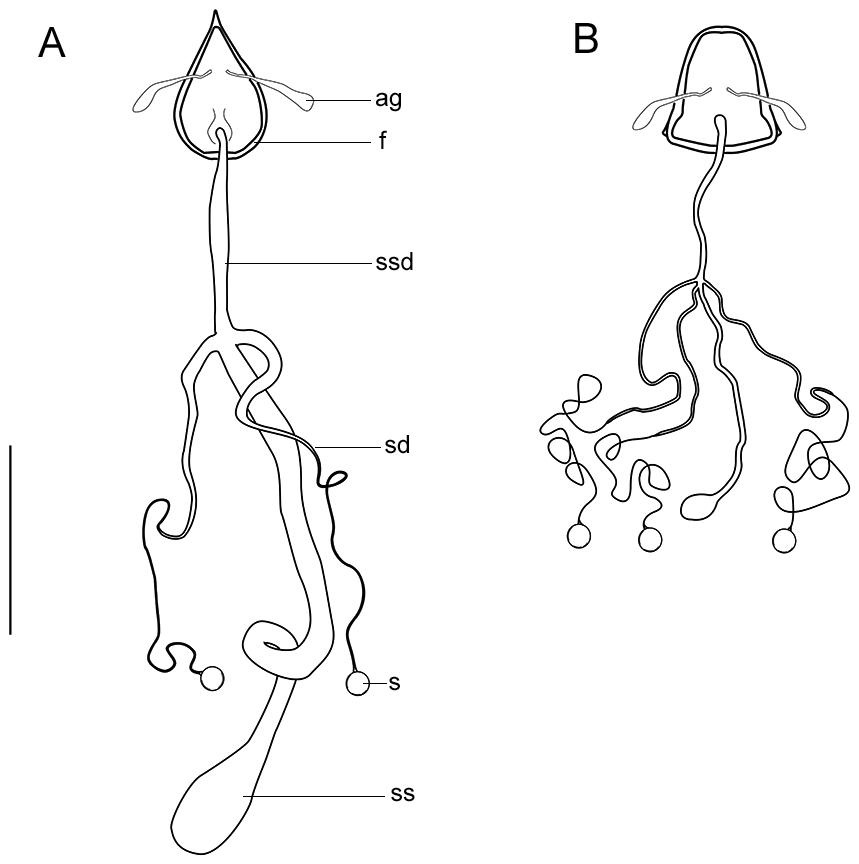
Distal reproductive complex. **A** *Zelothrix yeatesi* sp. n. **B** *Sidarena macfarlandi* sp. n. Scale line: 0.2 mm. Abbreviations: *ac*, accessory gland; *f*, furca; *s*, spermatheca; *sd*, spermathecal duct; *ss*, spermathecal sac; *ssd*, spermathecal sac duct.

### Sidarena aurantia sp. n.

Taxon classificationAnimaliaDipteraTherevidae

http://zoobank.org/89BA1398-15FC-4E48-AC36-0107EC85556B

Figs 5 , 6

#### Type material.

**Holotype** male, AUSTRALIA: Western Australia: 158 km S Newman, 9 km N Kumarina Roadhouse, Malaise in wide sandy wash, 21/23.V.2003, M.E. Irwin F.D. Parker, 638 m 24°37.8'S, 117°36.8'E (GPS) (ANIC).

**Paratypes**. AUSTRALIA: Western Australia: 13 males, same data as holotype (ANIC, CSCA); male, same data as holotype, 18/21.V.2003 (CSCA); male, 74 km S Newman on Great Northern Highway, Malaise in wash with drying pools, 6/18.V.2003, M.E. Irwin F.D. Parker, 631 m 23°56.0'S, 119°46.0'E (GPS) (ANIC).

#### Diagnosis.

Medium-sized fly with distinct yellow-orange colouration on body; pleuron base colour yellow-orange dorsally, blackish suffusion ventrally and on coxae; abdomen yellow-orange, sternites 1–3 with black suffusion laterally; postocular macrosetae relatively short; femora orange with black suffusion (variable); wing uniformly dark infuscate.

#### Description.

*Body length*. 8.0 mm (male). *Head*. (Fig. 5) Yellowish-grey pubescent; frons flat, brownish-orange pubescent, admixed with scattered black setae dorsolaterally and immediately above antennal socket, narrow dark pubescence present along eye margin; occiput convex with two rows of dark postocular setae, dark setae extending ventrally onto gena and admixed with finer white setae; antennal scape length less than pedicel and flagellum combined, orange-yellow pubescent with numerous robust black setae laterally; pedicel brownish-orange pubescent with numerous black setae; flagellum with extensive brown pubescence; mouthparts yellowish with dark pile. *Thorax*. Base colour dark yellow-orange on scutum and dorsally on pleuron; scutum overlain with pubescence as broad brown lateral stripes and broad metallic-grey stripe medially (some individuals with brownish suffusion anteromedially); very fine, sparse setal pile on scutum; postpronotal lobe orange pubescent; pleuron dark-yellow orange dorsally, darker suffusion ventrally and anteriorly; pleuron with fine white pile on anepisternum and katatergite; chaetotaxy: notopleural setae (np), 4; supra alar setae (sa), 2; postalar setae (pa), 1; dorsocentral setae (dc), 3; scutellar setae (sc), 1; wings dark infuscate, most cells slightly paler centrally; venation dark; coxae black; femora dark yellow-orange with variable extent of black suffusion, more pronounced on hind femur; tibiae and tarsi dark yellow-orange, distal tarsomeres blackish. *Abdomen*. Mostly dark yellow-orange, dark suffusion laterally on anterior sternites; silver velutum on tergites 1–5; sparse setal pile on all segments, short and dark dorsally, elongate and white laterally on anterior segments, setal pile denser and more robust posteriorly. *Male Terminalia*. (Fig. 6) Dark yellow-orange; epandrium numerous dark robust setal laterally, setal pile shorter medially; tergite 8 quadrangular with dark setae laterally; gonocoxite rounded with outer process well developed and rounded, extensive robust setal pile present; hypandrium triangular and fused with gonocoxites laterally; velutum pile very sparse ventrally on gonocoxites (barely evident in some cases); ventral lobe triangular; gonocoxite halves approximating medially, lacking medial atrium; inner gonocoxal process articulating dorsally on gonocoxite, curved medially and with few setae apically; gonostylus narrow and slightly curved medially, numerous setae midway along medial surface; aedeagus with dorsal apodeme of parameral sheath ‘T’-shaped, subequal in length to ventral apodeme; distiphallus broad distally with small spines apically.

#### Comments.

*Sidarena aurantia* sp. n. is known only from a series of males collected in Malaise traps in northern Western Australia. The yellow-orange abdomen is highly distinctive for this species, and it is very similar to *Sidarena macfarlandi* sp. n. There are subtle differences between the two species, which at this stage, based on the material examined, we recognise as separate species. The abdomen of *Sidarena aurantia* sp. n. is more uniformly orange (darker on anterior tergites in *Sidarena macfarlandi* sp. n.), while the lower half of the pleuron, coxae and hind femora are darker in *Sidarena aurantia* sp. n. The female is unknown for this species.

#### Etymology.

The specific epithet is a Latin adjective in the nominative feminine singular, meaning orange-coloured, referring to the body colouration.

### Sidarena flavipalpa sp. n.

Taxon classificationAnimaliaDipteraTherevidae

http://zoobank.org/6BBF2B14-6CB7-4127-965F-96D074D17C1D

Figs 7 , 8 , 9

#### Type material.

**Holotype** male, AUSTRALIA: Queensland: Beaudesert, 14.vii.1953, K.R.N., in cop. (CSCA) (MEI028781).

**Paratype**. AUSTRALIA: Queensland: female, same data and mounted with holotype (CSCA) (MEI028782).

#### Diagnosis.

Medium sized fly with light brownish-grey coloured thorax; abdomen base colour dark brown; scutum brown with light grey dorsocentral stripes; occiput overlain with tan-grey pubescence; wing slightly infuscate, darker along wing veins.

#### Description.

*Body length*. 6.5 mm (male), 7.0 mm (female). *Head*. Light brown-tannish grey pubescent; frons darker along eye margin (as a spot in female), admixed with sparse dark setae, especially above antennal socket; occiput convex with dark postocular setae not arranged in rows in either sex, setae of similar length in both sexes, dark setae extending onto gena where they are admixed with paler setae; antennal scape longer than pedicel and flagellum combined, distinctly thicker; scape yellow with brownish suffusion laterally, numerous robust dark setae laterally; pedicel brownish with dark setae; flagellum brown, conical, darker apically; mouthparts yellow with white setae. *Thorax*. Scutum with extensive dark brown matte pubescence, orange pubescence laterally and on postpronotal lobe, distinct light grey dorsocentral stripes along entire scutum length; scutal pile very fine and sparse; scutellum yellow with sparse grey pubescence; pleuron tan-grey pubescent with fine white setae confined to anepisternum and katatergite. Coxae yellow, overlain with grey pubescence; legs entirely yellow with black macrosetae; chaetotaxy: np, 4; sa, 2; pa, 1; dc, 4; sc, 1; wing hyaline with brownish tint. *Abdomen*. Tergites dark brown to black with yellow laterally, sternites yellow; all segments with sparse short setae, lateral setae white in male, especially on anterior segments. *Male terminalia*. (Fig. 9) Epandrium longer than wide, with medium length black setae over entire surface; hypoproct rounded apically; gonocoxite ovate, outer gonocoxal process elongate and rounded, reaching half the distance to the tip of the inner gonocoxal process; inner gonocoxal process slightly spatulate at apex, extending to tip of gonostylus; gonostylus with a brush of light brown setae along inner surface; gonocoxite with elongate dark brown setae on lateral surface, denser over area ventrad of outer gonocoxal process; aedeagus typical of other species in the genus. *Female terminalia*. Short white setae dorsally on 4/5^ths^ of tergite 8; tergite 8 longer than broad; sternite 8 setose surface thinly sclerotized, ovoid in shape, narrower posteriorly; six acanthophorite A1 setae strong, 11 longer, thinner A2 setae directed ventrally.

#### Comments.

*Sidarena flavipalpa* sp. n. is the only species of the genus found in eastern Australia. The species is only known from a pair collected in copula, and mounted together on the same pin; the holotype is the male, while the female is the paratype. This species is also distinctive by the scutal pubescent pattern, which is different from other species in the genus.

#### Etymology.

The specific epithet is a noun in apposition derived from combining the Latin adjective *flavus*, meaning yellow, and noun *palpus*, meaning feeler, referring to the colour of the palpi.

### Sidarena geraldton sp. n.

Taxon classificationAnimaliaDipteraTherevidae

http://zoobank.org/B7AD7460-2384-4D24-B9C3-583D771E6219

Figs 10 , 11

#### Type material.

**Holotype** male, AUSTRALIA: Western Australia: Geraldton, 7.vii.1972, N. McFarland (at light) (ANIC) (MEI028795).

#### Diagnosis.

Relatively small and slender species; scape grey pubescent, relatively narrow; pleuron uniform grey pubescent; abdomen dark brown-black; scutum with broad metallic grey stripe with medial brown suffusion; wing with faint infuscation, darker anteriorly and along veins; legs brown.

#### Description.

*Body length*. 5.5 mm (male). *Head*. Occiput silver-grey pubescent with two rows of elongate postocular setae; frons flat, dark brown pubescent admixed with elongate black setae, especially just above antennal socket; pubescence also as black line along eye margin and silver spot lateral to antennal socket; face silver pubescent; gena silver-white pubescent with fine, white, elongate setae; antenna as long as head length, scape similar width to pedicel and flagellum and as long as both combined, brown with grey pubescence with extensive black setae on outer surface; flagellum brown pubescent, tapered to dark arista; mouthparts dark brown with black setae. *Thorax*. Dark base colour overlain with extensive grey pubescence; scutum overlain with pubescence as broad brown lateral stripes and broad metallic-grey stripe medially with brown suffusion along axis; scutellum yellowish with grey pubescence; pleuron mostly grey pubescent, lacking setae except anepisternum and katatergite which have scattered short, white setae; coxae dark grey pubescent; legs uniformly brown with black setae; chaetotaxy: np, 4; sa, 1; pa, 1; dc, 3; sc 1; wing uniformly tinted infuscate, venation dark. *Abdomen*. Dark brown-black with cream-yellow areas laterally and on tergite 1, extensive silver velutum on at least tergites 1–4 in male, velutum not unidirectional, but with triangular pattern depending on angle viewed; thin setae present on all segments, black medially and posteriorly, white laterally and anteriorly. *Male terminalia*. (Fig. 11) Epandrium longer than wide, setae more robust laterally; cerci distinctly separate, ovoid; tergite 8 quadrangular with short robust setae laterally; gonocoxite wider than long; outer gonocoxal process elongate, pointed; inner gonocoxal process elongate, its apex protruding posteriorly to apex of gonostylus but more thinly sclerotized and slightly spatulate apically; gonostylus slender with scattered black setae anteriorly at about 1/3 distance from base to apex; ventral lobe broad and rounded; hypandrium connected to gonocoxite along anterior edge; aedeagus shape similar to other species in genus.

#### Comments.

*Sidarena geraldton* sp. n. is a western species known only from the male holotype collected from Geraldton, Western Australia. This is a relatively diminutive species with dark legs, abdomen and narrow wings.

#### Etymology.

The specific epithet is the unaltered place name of the type locality for this species; a noun in apposition.

### Sidarena hortorum sp. n.

Taxon classificationAnimaliaDipteraTherevidae

http://zoobank.org/B49F0340-28A9-413E-8A31-B250AE827F94

Figs 1 , 2 , 12 , 13

#### Type material.

**Holotype** male, AUSTRALIA: Western Australia: 37 km W Binnu, [-28.033, 114.667], 9.VII.1972, hand netted, N. McFarland. (ANIC) (MEI028783).

**Paratypes.** Two males, female, same data as holotype (ANIC, CSCA) (MEI028784, 028785, 028794); female, Cooralya H.S., [-24.45, 114.067], 10.IX.1971, hand netted, K. T. Richards. (WAM) (MEI028780); male, Gin Gin, 8 mile peg, [-31.35, 115.9], 17.VIII.1964, hand netted, P. Lawrence; 8 mile peg. (WAM) (MEI028779).

#### Diagnosis.

Medium sized flies; thorax yellowish dorsally on pleuron, darker ventrally, scutum with broad grey strip and narrow medial brown suffusion; abdomen dark brown, yellow laterally; wing hyaline, faintly infuscate anteriorly and along veins; legs yellowish with dark suffusion; male postocular setae variable in length but often elongate.

#### Description.

*Body length*. 8.0 mm (male), 9.0 mm (female). *Head*. (Figs 12, 13A) Yellowish-grey pubescent (male), silver-grey pubescent (female); frons flat, dark brownish pubescent, admixed with scattered black setae dorsolaterally and immediately above antennal socket, narrow dark pubescence present along eye margin; occiput convex with two poorly defined rows of dark postocular setae, dark setae extending ventrally onto gena and admixed with finer white setae; parafacial with yellow-silver pubescence; antennal scape length less than pedicel and flagellum combined, orange-silver pubescent, darker laterally, with numerous robust black setae laterally; pedicel brownish-orange pubescent with numerous black setae; flagellum with extensive dark brown pubescence; mouthparts yellowish with dark pile. *Thorax*. Base colour dark yellow-orange on scutum and posterodorsally on pleuron; scutum overlain with pubescence as broad brown lateral stripes and broad metallic-grey stripe medially (dark brownish suffusion along axis distinct in male); fine, sparse setal pile on scutum; postpronotal lobe orange pubescent; scutellum yellow; pleuron dark-yellow orange posterodorsally, darker suffusion ventrally and anteriorly, covered with dense grey pubescence; pleuron with fine white pile on anepisternum and katatergite; chaetotaxy: np, 3; sa, 2; pa, 1; dc, 4; sc, 1; wing hyaline with dark tinge, especially anteriorly and along wing veins; venation dark; coxae dark with grey pubescence; legs dark yellow, femora with distinct brown suffusion; distal tarsomeres black. *Abdomen*. Dark yellow, extensive black-brown area medially on all tergites; silver velutum on tergites 1–5 in male. *Male terminalia*. (Fig. 13B–E) Epandrium longer than wide, with brown marking medially and robust dark setae, longer laterally; cercus darker than epandrium; gonocoxites with outer gonocoxal process heavily sclerotized, pointed apically; inner gonocoxal process with few setae, spatulate apically; gonostylus narrow with setae midway along medial surface; ventral lobe bluntly rounded; aedeagus typical for genus. Female terminalia typical for genus.

#### Comments.

*Sidarena hortorum* sp. n. is a western species closely related to *Sidarena aurantia* sp. n. and *Sidarena macfarlandi* sp. n. based on body colouration and scutal pattern.

#### Etymology.

This species is a patronym named in honour of Fred and Jean Hort, field naturalists and photographers who enthusiastically document the flora and fauna of Western Australia.

### Sidarena macfarlandi sp. n.

Taxon classificationAnimaliaDipteraTherevidae

http://zoobank.org/2E3A2F57-634D-4DD0-871D-564B73BC3935

Figs 14 , 15 , 16 , 28B

#### Type material.

**Holotype** male, AUSTRALIA: Western Australia, Moresby Range, 12.9 km NE Geraldton, Mills Park, [-28.660, 114.661], 1.viii.1973, hand netted, N. McFarland. (MEI028790) (ANIC).

**Paratypes.** AUSTRALIA: Western Australia: 2 males, female, same data as holotype (MEI028791, 028255 [male in copula], 028256 [female in copula]) (ANIC); males, same data as holotype (MEI028787); 6 males, 2 females, Moresby Range, Howatharra Rd., [-28.54, 114.667], 1.viii.1974, black light (UV), N. McFarland. (ANIC, CSCA) (MEI028788, 028789, 129016, 028257, 028792, 028793, 129014, 028254); female, Greenough, [-28.95, 114.733], 29.viii.1978, hand netted, R. P. McMillan. (WAM) (WAM872094).

#### Diagnosis.

Abdomen distinctly orange; wing slightly mottled; legs dark yellow with brown suffusion on hind femur; pleuron dark yellow dorsally; abdominal tergite 1 with dark brown markings.

#### Description.

*Body length*. 7.0 mm (male), 8.0 mm (female). *Head*. (Fig. 14–15) Yellowish-grey pubescent; frons flat, brownish-orange pubescent, admixed with scattered black setae dorsolaterally and immediately above antennal socket, narrow dark pubescence present along eye margin; occiput convex with dark postocular setae not arranged in rows, dark setae extending ventrally onto gena and admixed with finer white setae; face yellow-grey pubescent; antennal scape length less than pedicel and flagellum combined, orange-yellow pubescent with numerous robust black setae laterally; pedicel brownish-orange pubescent with numerous black setae; flagellum with extensive brown pubescence; mouthparts yellowish with dark pile. *Thorax*. Base colour dark yellow-orange on scutum and dorsally on pleuron; scutum overlain with pubescence as broad brown lateral stripes and broad metallic-grey stripe medially (with light yellow-brownish suffusion medially and narrow dark stripe along axis); very fine, sparse setal pile on scutum; postpronotal lobe orange pubescent; pleuron dark-yellow orange dorsally, darker suffusion ventrally, pleuron with fine white pile on anepisternum and katatergite; chaetotaxy: np, 3–4; sa, 2; pa, 1; dc, 3; sc, 1; wings infuscate, darker along veins, more distinctive and extensive around crossveins to give mottled appearance; venation dark; coxae dark, overlain with grey pubescence; femora dark yellow-orange with dark suffusion more pronounced on hind femur; tibiae and tarsi dark yellow-orange, distal tarsomeres blackish. *Abdomen*. Mostly dark yellow-orange, dark suffusion laterally on segments 1–3 and sometimes segment 4; silver velutum on tergites 1–6; sparse setal pile on all segments, short and dark dorsally, elongate and white laterally on anterior segments, setal pile darker and more robust posteriorly. *Male terminalia* (Fig. 16). Epandrium slightly longer than wide with extensive robust dark setae; cercus with pale setae; tergite 8 slightly emarginate posteriorly; gonocoxite and aedeagus typical of species in the genus. Female terminalia typical for the genus.

#### Comments.

*Sidarena macfarlandi* sp. n. is similar in appearance to *Sidarena aurantia* sp. n. based on body colouration, especially the abdomen (see comments above).

#### Etymology.

This species is named after the collector, Noel McFarland, of this and other species of *Sidarena* in Western Australia.

### Sidarena yallingup sp. n.

Taxon classificationAnimaliaDipteraTherevidae

http://zoobank.org/1FD0C2F9-7A52-4E00-A5B3-0A3A64378F34

Figs 17 , 18 , 19 , 20

#### Type material.

**Holotype** male, AUSTRALIA: Western Australia, 37 km N Augusta, [-34.333, 115.167], 1.x.1975, hand netted, K. A. Spencer. (WAM872079) (WAM).

**Paratypes.** AUSTRALIA: Western Australia: male, 2 females, Leeuwin Naturaliste National Park, Yallingup portion, 14.ix.1983, hand netted, E. I. Schlinger, M. E. Irwin; limestone caves and Acacia-Eucalyptus forest, cycads (CSCA) (MEI028771, 028772, 028773); 2 females, Hamelin Bay, [-34.2, 115.017], 1.x.1975, hand netted, K. A. Spencer. (WAM872077, 872078) (WAM); female, Hamelin Bay, [-34.2, 115.017], 2m, 26.ix.1962, hand netted, E. S. Ross, D. Q. Cavagnaro (CASC) (MEI028774).

#### Diagnosis.

Wing dark mottled infuscate; scutal macrosetae elongate, legs dark brown; abdomen dark brown; genal pile uniformly dark.

#### Description.

*Body length*. 7.5 mm (male), 8.5 mm (female). *Head* (Fig. 17–18). Grey-silver (male) or yellow-silver (female) pubescent; male occiput convex with silver-grey and matte black pubescence (depending on angle viewed); postocular setae not arranged in rows, setae variable in length, some elongate; frons flat with elongate black, scattered setae, at midpoint of the frons is a small dark patch of dark brown pubescence set against eye margin; gena with dark setae; face golden (female), silver (male) pubescent, without setae; antennae light yellowish-brown; palpus brown with dark setae; antenna light yellow-orange, scape wider than pedicel and flagellum, with dark setae on outer lateral surface, longer than pedicel and flagellum combined; flagellum orange, tapered to a dark brown arista. *Thorax*. Scutum with distinct matte black (gold when viewed laterally) stripes laterally, broad medial stripe greyish in male, yellowish-grey in female, narrow dark brown suffused stripe along axis in male; postpronotal lobe orange; scutal macrosetae elongate, black, sparse thin scutal pile otherwise; pleuron dark yellow, darker ventrally and on coxae, with sparse covering of grey pubescence; sparse elongate, thin black setae on anepisternum and katatergite; scutellum pale yellow-orange with grey pubescence; chaetotaxy: np, 3; sa, 2; pa, 1; dc, 6–7; sc 2; wing dark mottled infuscate; coxae dark brown with grey pubescence; femora dark brown; rest of legs light brown. *Abdomen*. Dark brown, dorsally black with silver velutum on tergites 2–5 (more extensive in male), velutum pattern not unidirectional and silver pattern changes depending on angle viewed; anterolaterally on tergite 1 is a small patch of orange. *Male* (Fig. 19) *and female* (Fig. 20) *terminalia*. Similar to other members of the genus.

#### Comments.

The mottled wing of *Sidarena yallingup* sp. n. is highly distinctive and easily identifies it among the other species with the metallic grey stripe on the scutum. There is more sexual dimorphism in this than in other species. This species appears to be closely related to *Sidarena geraldton* sp. n. based on overall body colouration and wing pattern.

#### Etymology.

The specific epithet is the unaltered place name Yallingup (which is an Aboriginal word meaning ‘Place of Love’) for a location in southwestern Western Australia where this species was collected; a noun in apposition.

### Zelothrix gen. n.

Taxon classificationAnimaliaDipteraTherevidae

http://zoobank.org/AF6D8499-EF68-4415-A8B4-4F9DAE431A6A

Figs 3 , 21 , 22 , 23 , 24 , 25 , 26 , 27 , 28

#### Type species.

*Zelothrix warrumbungles* sp. n., designated here.

#### Diagnosis.

Male eyes contiguous dorsally; male occiput concave with a single row of postocular macrosetae present dorsally in male; antennal scape with macrosetae along medial surface; scape narrow and only slightly elongate; two pair of scutellar macrosetae; parafacial without setal pile; velutum patches absent on femora and sparsely present ventrally on gonocoxites; single anteroventral seta present apically on hind femur; wing cell m_3_ open; male genitalia with inner gonocoxal process well developed; gonostylus narrow apically; gonocoxites with medial atrium lacking; aedeagus with distiphallus broad apically; ventral apodeme of parameral sheath as broad plate, not forked; epandrium quadrangular. Female tergite 8 with narrow process anteromedially; two spermathecae, ducts joining to spermathecal sac duct; spermathecal sac present (Fig. 28A), female abdominal segment 8 with elongate posteriorly directed setae (Fig. 27).

#### Included species.

*Zelothrix warrumbungles* sp. n. and *Zelothrix yeatesi* sp. n.

#### Comments.

*Zelothrix* gen. n. is a distinctive genus with a disparate distribution. Similar genera include *Squamopygia* Kröber, *Taenogerella* Winterton & Irwin and *Sidarena* gen. n. This new genus can be differentiated from *Squamopygia* and *Sidarena* gen. n. by the presence of two scutellar macrosetae and a medial atrium in the male gonocoxites. The wing is extensively patterned in *Zelothrix* gen. n. but not banded as in *Squamopygia*. *Zelothrix* gen. n. is separable from *Taenogerella* by the latter having a downward directed distiphallus in the male genitalia and three spermathecae (two in *Zelothrix* gen. n.). A significant departure from the female genitalic complement of three spermathecae typically found in Agapophytinae, is that *Zelothrix* gen. n. only has two spermathecae, a condition found in Therevinae. No other genus of Agapophytinae has two spermathecae, although the distantly related agapophytine genus *Bonjeania* Winterton & Skevington has only a single spermatheca (Winterton et al. 2000).

*Zelothrix warrumbungles* sp. n. is a locally highly abundant species found mainly in Warrumbungle National Park (New South Wales) (Fig. 4), while *Zelothrix yeatesi* sp. n. is a rarely collected species endemic to Porongurup National Park (Western Australia). The two species are very similar in appearance.

#### Etymology.

This name is derived from the Greek, *Zelos*– emulation, and *thrix*– hair, for the setal pile on the female abdomen. Gender is feminine.

#### Key to species of *Zelothrix* gen. n.:

**Table:** 

1	Forefemur dark brown; male frons predominantly silver pubescent immediately above antennal socket; antenna greater than 1.5× head length (Figs 3, 25–27) (Eastern Australia)	***Zelothrix warrumbungles* sp. n.**
–	Forefemur dark yellow; male frons black and silver pubescent immediately above antennal socket; antenna less than 1.5× head length (Figs 21–24) (Western Australia)	***Zelothrix yeatesi* sp. n.**

### Zelothrix warrumbungles sp. n.

Taxon classificationAnimaliaDipteraTherevidae

http://zoobank.org/778C5F21-B145-4EB6-B6A6-899D650F0CB3

Figs 3 , 21 , 22 , 23

#### Type material.

**Holotype** male, AUSTRALIA: New South Wales: Warrumbungle National Park, 1.7 km N Camp Blackman, Buckleys Creek, [-31.25, 149.002], 480m, 30.x.-14.xi.1997, malaise trap, S. Winterton, J. Skevington. (ANIC) (MEI153269).

**Paratypes.** AUSTRALIA: New South Wales: 22 males, 10 females, same data as holotype, (ANIC) (MEI140101, MEI140126, MEI140128, MEI140131, MEI140138, MEI140141, MEI140143, MEI140150, MEI140153, MEI140159, MEI140375-95, MEI153269). Queensland: female, Stanthorpe, [-28.667, 151.917], 10.i.1924, hand netted, F. M. Hull. (CNC) (MEI027295); female, near Stanthorpe, Mount Marlay, [-28.667, 151.933], 1.x.1987, hand netted, D. K. Yeates. (QM) (MEI033880). Victoria: 24.2 km NNE Orbost, [-37.75, 148.5], 5.xi.1969, hand netted, I. F. B. Common. (ANIC) (MEI028778).

#### Diagnosis.

Forefemur dark brown; male frons predominantly silver pubescent immediately above antennal socket; antenna greater than 1.5x head length.

#### Description.

*Body length*. 7.5 mm (male), 8.5 mm (female). *Head*. (Figs 21–22) Silver-grey pubescent; ocellar tubercle black, raised (prominent in male); frons flat, with only a few black setae above the antennal socket, silver and black pubescent in male, matte black, silver and gold patterned in female; occiput silver-gold pubescent, concave with a single row of black postocular setae dorsally in male, two rows in female; gena silver pubescent admixed with pale setae; parafacial silver in male, silver and matte black in female; palpus narrow, pointed apically, with brown with black setae;. Antennal scape elongate and cylindrical, orange, with erect black setae on all surfaces, slightly thinker than flagellum and length equalling length of combined pedicel and flagellum; flagellum elongate and cylindrical, brown pubescent with distinct angled arista at apex. *Thorax*. Scutum and scutellum gold-silver pubescent, scutum with three distinct dark brown stripes, medial stripe extending onto scutellum; chaetotaxy: np, 4; sa, 2; pa, 1; dc, 3; sc, 2; pleuron base colour black, overlain with dense greenish-silver pubescence extending onto coxae; thin white hairs on anepisternum and katatergite; femora bright yellow, forefemur mostly with dark brown to black suffusion; tibiae and tarsomeres 1 and 2 dark yellow, brown apically; remaining tarsomeres dark brown; wing distinctly infuscate with extensive mottled pattern; venation dark. *Abdomen*. Slender, elongate, bright yellow with small dark brown area anteromedially on tergites in male, tergites more extensively dark brown in female and overlain with sparse grey pubescence; sparse thin elongate setae on all segments, mostly pale, but darker dorsomedially and on terminalia. *Male Terminalia*. (Fig. 23) Epandrium longer than wide, slightly tapered posteriorly, sclerotised posterolaterally, setae sparse, more elongate laterally; cercus relatively small; gonocoxites rounded with short round outer gonocoxal process; hypandrium small and fused to gonocoxites anteriorly; gonocoxal apodemes small and rounded; setae on gonocoxites sparse, elongate and erect, with sparse velutum ventrally on gonocoxite; inner gonocoxal process and gonostylus narrow and curved medially; ventral lobe elongate and rounded apically; dorsal apodeme of parameral sheath ‘T’-shaped; ventral lobe broad, not forked and projecting beyond dorsal apodeme; distiphallus broad, irregularly shaped dorsally, small spines apically; lateral ejaculatory apodemes narrow and angled posteriorly, basiphallus small. *Female terminalia*. Similar to the other species in this genus.

#### Comments.

*Zelothrix warrumbungles* sp. n. is a distinctive, elegant and abundant species in the type locality during the late summer months, and in some years may be the most commonly encountered species of stiletto fly during this time.

#### Etymology.

The specific epithet is the unaltered place name Warrumbungles (which is an Aborignal name for this mountain range, meaning “crooked mountains”) referring to the mountain range where this species was collected; a noun in apposition.

### Zelothrix yeatesi sp. n.

Taxon classificationAnimaliaDipteraTherevidae

http://zoobank.org/38B927CB-50F3-44F0-85FA-FEB0B0F25A1C

Figs 24 , 25 , 26 , 27 , 28

#### Type material.

**Holotype** male, AUSTRALIA: Western Australia, Porongurup National Park, [Porongurup Range], Yate Flats, [-34.667, 117.85], 11.xi.1987, malaise trap, M. E. Irwin, E. I. Schlinger (ANIC) (MEI028776).

**Paratypes.** AUSTRALIA: Western Australia: female, same data as holotype (ANIC) (MEI028776); male, Porongurup National Park, [Porongurup Range], Jarra-Karri Forest, Mira Flores Hut, [-34.667, 117.85], 11.xi.1987, hand netted, M. E. Irwin, E. I. Schlinger. (CSCA) (MEI028775).

#### Diagnosis.

Forefemur dark yellow; male frons black and silver pubescent immediately above antennal socket; antenna less than 1.5x head length.

#### Description.

*Body length*. 6.0 mm (male), 6.5 mm (female). (Figs 24–25) Similar to *Zelothrix warrumbungles* sp. n. except as follows: Antenna shorter, scape slightly wider; frons of male with matte black pubescence more extensive above antennae socket; scutal chaetotaxy: np, 4; sa, 1–2; pa, 1; dc, 4; sc, 2; foreleg dark yellow; male abdominal tergites with more extensive dark markings medially. *Male* (Fig. 26) *and female* (Fig. 27, 28A) *terminalia*. Very similar to the other species in this genus. Female tergite 8 with anterior process relatively narrow; broad band of elongate setae directed posteriorly on both tergite 8 and sternite 8; furca broadly tear-drop shaped

#### Comments.

*Zelothrix yeatesi* sp. n. is very similar in body colour and wing patterning to *Zelothrix warrumbungles* sp. n., but is much less commonly collected. The shape and vestiture of the frons and antennal shape differentiate this species.

#### Etymology.

This distinctive species is a patronym named in honour of our colleague, friend and oft mentor, Dr David K. Yeates.

## Supplementary Material

XML Treatment for Sidarena

XML Treatment for Sidarena aurantia

XML Treatment for Sidarena flavipalpa

XML Treatment for Sidarena geraldton

XML Treatment for Sidarena hortorum

XML Treatment for Sidarena macfarlandi

XML Treatment for Sidarena yallingup

XML Treatment for Zelothrix

XML Treatment for Zelothrix warrumbungles

XML Treatment for Zelothrix yeatesi
